# Mechanism of Case Processing in the Brain: An fMRI Study

**DOI:** 10.1371/journal.pone.0040474

**Published:** 2012-07-12

**Authors:** Satoru Yokoyama, Hideki Maki, Yosuke Hashimoto, Masahiko Toma, Ryuta Kawashima

**Affiliations:** 1 Institute of Development, Aging, and Cancer, Tohoku University, Sendai-city, Japan; 2 Faculty of Regional Studies, Gifu University, Gifu-city, Japan; 3 Akita International University, Yuwa, Akita-city, Japan; Baycrest Hospital, Canada

## Abstract

In sentence comprehension research, the case system, which is one of the subsystems of the language processing system, has been assumed to play a crucial role in signifying relationships in sentences between noun phrases (NPs) and other elements, such as verbs, prepositions, nouns, and tense. However, so far, less attention has been paid to the question of how cases are processed in our brain. To this end, the current study used fMRI and scanned the brain activity of 15 native English speakers during an English-case processing task. The results showed that, while the processing of all cases activates the left inferior frontal gyrus and posterior part of the middle temporal gyrus, genitive case processing activates these two regions more than nominative and accusative case processing. Since the effect of the difference in behavioral performance among these three cases is excluded from brain activation data, the observed different brain activations would be due to the different processing patterns among the cases, indicating that cases are processed differently in our brains. The different brain activations between genitive case processing and nominative/accusative case processing may be due to the difference in structural complexity between them.

## Introduction

As a higher cognitive function, human language has a unique grammatical rule system, namely syntax, which interplays with the speech sound processing system (phonology) and the meaning processing system (semantics) [1], [2]. This rule system enables us to create and understand an infinite number of sentences by using various combinations of words, and is assumed to consist of several subsystems.

The case system is one such subsystem, and it plays a crucial role in signifying the relationships between noun phrases (NPs) and elements in sentences with which the NPs are closely related such as verbs, prepositions, nouns, and tense. Some languages, such as Japanese, attach case particles (e.g., –*ga* [nominative] and –*o* [accusative]) to NPs, while others, such as English, use morphological case markers that inflect pronouns in order to show their roles within a sentence. For example, a simple sentence consists of one predicate and more than one argument (or NP) as is seen in such sentences as *She saw him* (in English) and *Kanojo-ga kare-o mita* (in Japanese), meaning “She saw him,” where *kanojo-ga*  =  she, *kare-o*  =  him, and *mita*  =  saw. However, the forms of NPs in a sentence are not free form, as demonstrated by the following sentences, which are totally unacceptable in English and Japanese, respectively: *She saw he* (in English) and *Kanojo-ga kare-ga mit*a (in Japanese). The unacceptability of these examples seems to originate from the form of the second occurrence of the NP. The NPs in the object position do not bear the object case or the accusative case, but instead bear the subject case or the nominative case. The fact that native language speakers immediately notice the unacceptability of such sentences clearly shows that the grammatical rule system of the human language contains a case system. Because a minor error in the case array of a given sentence results in an ungrammatical sentence (as shown above), the case system, which regulates proper case array, must be crucial to human language processing. This is assumed in any theory of language.

The major cases belonging to the case system are the nominative, accusative, and genitive cases. The nominative case co-occurs with the tense of a sentence, the accusative case with a transitive verb, and the genitive case with a noun. Although these three types of cases exist independently in the human language, there are theoretically two ways to group them. One way is to group the nominative and genitive cases because they both can appear in the subject position, as in *he claims that*… and *his claim that*…, for example. The same situation is also observed in different languages; for example, in Japanese, in *kare-ga/-no iru heya* ‘the room where he is’, the subject *kare* ‘he’ allows the alternation between the nominative and genitive cases [3], [4]. Hence, such a dissociation may exist in our brain. The other way is to group the nominative and accusative cases because they both appear in a tensed sentence, as in *she saw him*, while the genitive case only appears within a NP, as in *his car*. Also, in theoretical linguistics, Chomsky (1986) [5] proposes a dichotomy of the types of cases based on the notions of structural case and inherent case, revising his original proposal about case in Chomsky (1981) [6]. In Chomsky (1981) [6], nominative, accusative, and genitive cases are all assigned under the same structural relation called “government” between the case assigner and the case assignee (NP), and all these cases are called “structural” cases. Chomsky (1986) [5] distinguishes the genitive case from the nominative and accusative cases, and categorizes the former as an “inherent” case, not a “structural” case, based on the observation that the NP *the city* that appears in the complement position of the noun *destruction* in (1a), below, receives case in two different ways; that is, by *of*-insertion in (1b) and by movement of the NP into the pre-nominal position and use of the genitive assignment rule in (1c), despite the fact that the NP is a logical object of the noun in both cases:

1. a. the destruction the city

- b. the destruction of the city
- c. the city’s destruction *t*

Chomsky (1986) states that inherent case is associated with θ-marking (i.e., close semantic linking between the case assigner [a noun, in this case] and the NP), while structural case is not, as shown in (2)-(3):

1. Nominative case assignment
  He seems [*t* to be smart].
  cf. It seems [that he is smart].
2. Accusative case assignment
  Mary believes [him to be smart].
  cf. Mary believes [that he is smart].

In (2), the pronoun *he* is a logical subject of the predicate *(to be) smart*, but cannot be assigned by it the nominative case. Thus, it has to move to the higher clause, where it is assigned the nominative case by the tense element on the predicate *seems*, which has no semantic linking with it. Likewise, in (3), the pronoun *him* is a logical subject of the predicate *(to be) smart*, but cannot be assigned by it the accusative case. Thus, it is assigned the accusative case by the verb *believes* in the higher clause, which has no semantic linking with it. Therefore, in Chomsky’s (1986) system [5], the genitive case is an “inherent” case, while the nominative and accusative cases are “structural” cases. Again, such a functional grouping may be represented in our brain.

Based on the above arguments regarding the nature of case, we would like to test whether the processing of the three types of cases in our brain differs between them or not. Although several neuroimaging studies have used case-related stimuli in their experimental designs (e.g., an agreement error between the subject and the verb; [7]), to our knowledge, there is only one neuroimaging study that directly examines case processing [8]. This study reported left inferior frontal activation as the common region for the processing of several cases. We can therefore expect the involvement of the left inferior frontal gyrus in case processing for sentences as well. However, because this previous study focused on case particles presented in an isolated situation rather than in a sentence context, it remains unclear how case is processed during sentence processing. Additionally, based on previous neuroimaging studies on sentence comprehension [9]–[16], we expected to find a difference in brain activity for the processing of cases in the left triangular and opercular part of the inferior frontal gyrus (Broca’s area), the left middle frontal gyrus, the left posterior part of the superior and middle temporal gyrus (Wernicke’s area), and the inferior parietal lobule.

## Methods

Fifteen native speakers of English (6 females aged 20–26 years; mean age 21.0 years) participated in this study. All participants had moved to Japan from the USA to study the Japanese language. All participants were right–handed, as confirmed by the Edinburgh Handedness Inventory [16]. None of the participants displayed any signs or had any previous medical history of diseases, including neurological diseases. Written informed consent in accordance with ethical committee of Medical School in Tohoku University and the Helsinki Declaration of Human Rights 1975 was obtained from each subject. This study was approved by ethical committee of Medical School in Tohoku University.

Three sets of conditions were prepared for the experiment: (1) the nominative case condition, (2) the accusative case condition, and (3) the genitive case condition. Sets (1), (2), and (3) each consisted of 28 sentences. All of the stimulus sentences were simplex sentences. In each stimulus sentence, either the nominative, accusative, or genitive form of a pronoun was replaced with a “-”. Under the stimulus sentence, three choices were displayed (e.g., 1. My, 2. Me, and 3. I). Examples of the stimuli are shown in Table 1. The order of choices was randomized across trials. Frequency, imageability, number of syllables, and number of letters in the words across conditions were controlled using the MRC Psycholinguistic Database (http://www.psy.uwa.edu.au/mrcdatabase/uwa_mrc.htm). There was no statistical difference among these three conditions (ANOVA: frequency, *p* = 0.464; the number of syllables, *p* = 0.264; and the number of letters, *p* = 0.329). In cases where it is too hard to match lexical items across conditions, this method is often used [11], [13].

**Table 1:** Examples of experimental stimuli.

Nominative case condition		
Stimulus sentence	- cooked the meals.	
Choices	1. My 2. Me 3. I	
**Accusative case condition**		
Stimulus sentence	The boss employed -.	
Choices	1. them 2. their 3. they	
**Genitive case condition**		
Stimulus sentence	- instructor corrected essays.	
Choices	1. Us 2. We 3. Our	

Both stimulus sentences and choices were presented at once in two lines.

In the fMRI experiment, the entire simplex sentences were visually presented. The visual stimuli were presented on the screen inside the MRI scanner for 3s, followed by presentation of a fixation cross for 3s. The inter-trial interval was set at 6s. Participants were asked to judge which choice was the correct choice by pressing buttons with their right hand. Trials were presented randomly. The accuracy rates and response times for all tasks were collected using a Windows–based computer. Visual presentation of the experimental stimuli was also performed using a Windows–based computer.

The fMRI scans were collected at Tohoku University on a 3T Intera Achieva scanner (Philips). Head motion was minimized by using cushions and tape around the subject’s head. Thirty axial slices (4 mm-thickness; FOV = 192 mm; data matrix, 64×64 voxels) were acquired every 2s during functional measurements [BOLD-sensitive gradient EPI sequence; TR = 2000 ms; TE = 30 ms; flip angle = 70°]. After functional image acquisition, anatomical images of the T1-weighted images were also acquired from all participants.

The fMRI time-series data were analyzed using SPM5 software (Wellcome Institute of Cognitive Neurology, http://www.fil.ion.ucl.ac.uk/) and implemented on MATLAB (Mathworks, Inc., Shelborn, Mass., USA). Slice timing adjustment, realignment, spatial normalization to the standard brain space, and smoothing with an isotropic Gaussian kernel of 8-mm full width at half-maximum using the standard SPM method were performed, and a high-pass frequency filter (128s) was applied. Time series were modeled and convolved using the hemodynamic response function. Contrasts among (1) nominative, (2) accusative, and (3) genitive case conditions were computed for each subject. For the above analysis, we used only correct trials. The group effects were computed using these contrast images with a random effect model (ANOVA). In this group effects analysis, we used reaction times as covariates to reduce the effect of any difference in reaction times among the conditions from brain activation results. A threshold was set at p<0.05 for multiple comparisons using whole-brain familiar wise error (FWE) correction.

In addition to making direct comparisons among the conditions, a post-hoc ROI analysis based on signal intensity was carried out in order to ascertain in detail how the detected regions were activated in each condition. We defined ROIs as the significantly activated clusters in direct comparisons among the three conditions. Mean parameter estimates in each ROI for each subject in each condition were calculated; ANOVAs were conducted for all conditions, and a post-hoc multiple comparison was performed (Bonferroni correction). The data on the accuracy rate and response time for all tasks were also examined by ANOVA and post-hoc multiple comparison (Bonferroni correction). Also, to test whether each region is activated for each condition, we used a one-sample t-test.

## Results

A statistically significant difference was seen in the accuracy rates among the three conditions (ANOVA: *p* = 0.004). In the post-hoc multiple comparisons, the genitive condition showed greater accuracy than the nominative and accusative conditions. Also, there was a statistically significant difference in the response times among the three conditions (ANOVA: *p* = 0.000). In the post-hoc multiple comparisons, the genitive condition showed longer response times than the others. The observed accuracy rates and response times are shown in Table 2.

**Table 2:** Behavioral data.

	Nominative	Accusative	Genitive
Accuracy rate (SD)	91.9% (0.04)	93.3% (0.03)	96.2% (0.04)
Response time (SD)	2325 ms (800)	2279 ms (691)	2750 ms (924)

**Figure 1:**
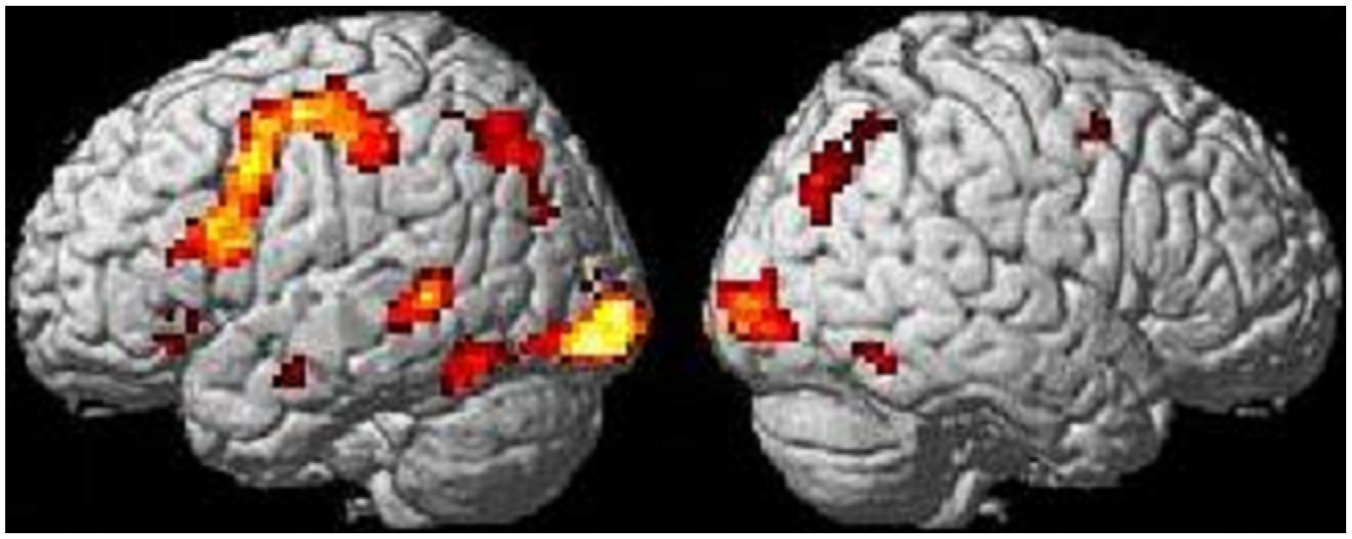
Commonly activated regions for the processing of all cases. The left and right figures show the left and right hemispheric activation results, respectively. A statistical threshold was set at p<0.05, FWE corrected.

**Figure 2:**
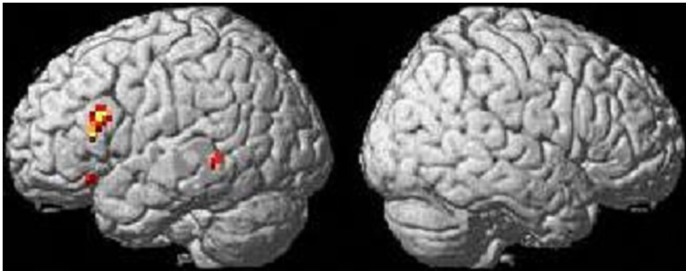
Different brain activations for processing among cases: ANOVA. The left and right figures show the left and right hemispheric activation results, respectively. A statistical threshold was set at p<0.05, FWE corrected.

**Figure 3:**
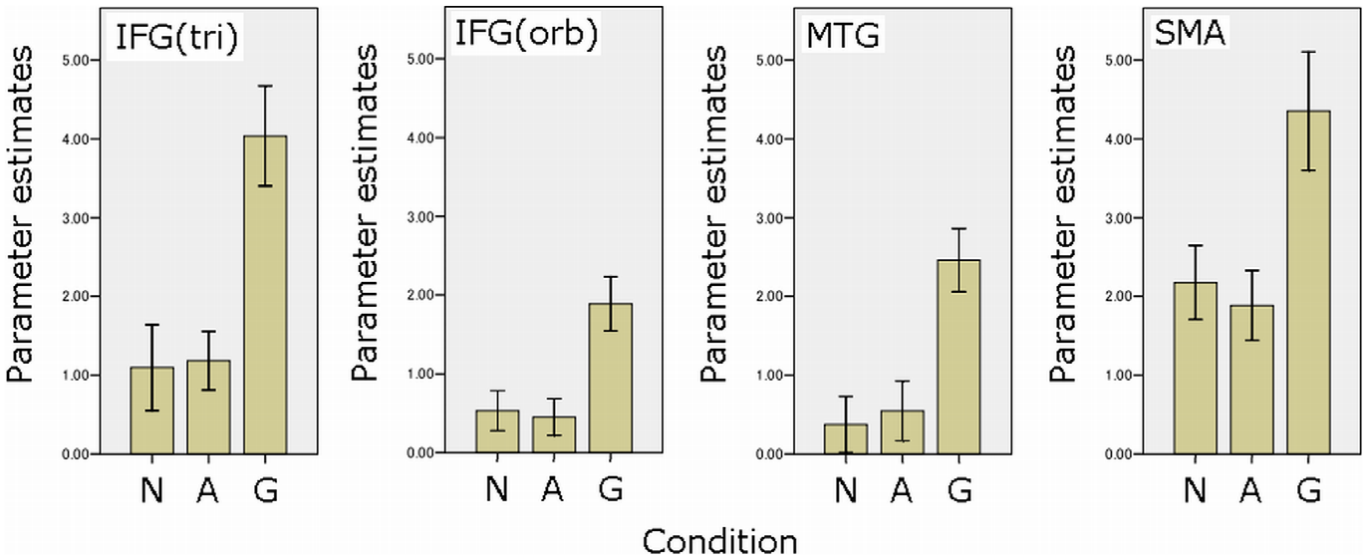
Results of regions of interest (ROI) analyses. These figures show the results of the left inferior frontal gyrus (triangular part)/IFGtri, inferior frontal gyrus (orbital part)/IFGorb, middle temporal gyrus/MTG, and supplementary motor area/SMA, respectively. N, A, and G denote the nominative case, accusative case, and genitive case conditions, respectively. In all results, the genitive case condition showed statistically greater activation than nominative and accusative case processing (Bonferroni correction, p<0.05).

In group analysis, all of the three conditions commonly activated the left triangular, opercular, and orbital parts of the inferior frontal gyrus and the posterior part of the superior/middle temporal gyri, as well as the bilateral inferior parietal lobule and inferior temporal gyrus, as shown in Fig. 1. In ANOVA with reaction times as a covariate, there was a robust statistical difference of brain activation among conditions in the left triangular and orbital parts of the inferior frontal gyrus, the posterior part of the middle temporal gyrus, and the supplementary motor area, as shown in Fig. 2. These results are summarized in Table 3. In post-hoc ROI analysis, for the above four regions, a genitive condition showed greater activation than nominative and accusative conditions, while there was no difference in brain activity between nominative and accusative conditions (Bonferroni correction, p<0.05). In the one-sample t-test for each region for each condition, while only nominative and accusative case conditions of the left middle temporal gyrus showed no statistically significant increase of activation (p = 0.15 and p = 0.085, respectively), others showed a statistically significant increase of activation (p<0.05). The results of these post-hoc ROI analyses are shown in Fig. 3.

**Table 3:** Results of brain activation among conditions.

Anatomical label	L/R	F	Z	x	y	z
Inferior frontal gyrus (tri)	L	24.81	5.29	−45	18	21
	L	24.06	5.22	−48	24	15
	L	23	5.13	−54	18	27
Inferior frontal gyrus (orb)	L	22.54	5.09	−33	27	−12
Middle temporal gyrus	L	22.42	5.08	−57	−45	−3
Supplementary motor area	L	21.91	5.03	−6	18	45

Abbreviations: tri; triangular part, orb; orbital part, L/R; left/right hemisphere.

Statistical threshold was set at p<0.05 FWE corrected.

## Discussion

In the current study, using fMRI, we investigated case processing in English by native English speakers. Our results showed that genitive case processing required greater brain activity in the left triangular part and orbital part of the inferior frontal gyrus, the posterior part of the middle temporal gyrus, and the supplementary motor area than nominative and accusative case processing (Fig. 2 and Table 3). Contrastively, between nominative and accusative case processing, there was no statistical difference in brain activation. Here, we would first like to discuss how the results we obtained in the current fMRI study are interpreted.

Experimentally, several confounding factors were involved in interpreting the results. First, the positions of the NPs in a sentence may be determined based on their case morphology, which in turn might affect the fMRI results. In fact, we were unable to completely control this effect in our experiment due to the nature of the English sentence structure, which did not allow for word order rearrangement. However, one piece of evidence suggests that this concern had no effect on the fMRI data in terms of the reaction times. In the task design, the accusative case was placed at the end of the stimulus sentences, while the nominative and genitive cases were placed at the beginning. Hence, if the word order had some effect on this experimental task, participants would have performed the trials in the nominative and genitive case conditions faster than those in the accusative case condition. However, results showed that the reaction times for trials in the nominative and accusative case conditions were in fact slower than those in the genitive case condition. Therefore, the assumption that the difference in the positions of NPs with a particular case affected the fMRI results does not hold. It is well known that the frequency, number of letters, and the number of syllables in words affect reaction times. As a second confounding factor, one may argue that variations in this frequency across tests might have affected the fMRI results. The characteristics of the stimuli, such as frequency, the number of letters, and the number of syllables in the words, were therefore controlled across conditions (see Methods). This method has been used in previous studies [11], [13]. Because there was no obvious statistical difference among the conditions for all characteristics, we conclude that the different stimuli showed no effect on the fMRI results across conditions.

Next, let us discuss how case is processed in our brain. Actually, our results showed a dissociation of genitive case processing from nominative and accusative case processing in brain activity, since a genitive case condition activated the left triangular and orbital parts of the inferior frontal gyrus, the posterior part of the middle temporal gyrus, and the supplementary motor area more than nominative and accusative case conditions did (Figs. 2 and 3). Based on these results, we found no evidence to support a grouping of the nominative and genitive cases in our brain, since there was no brain region more greatly activated in both nominative and genitive conditions than the accusative condition (see Figs. 2 and 3). Also, our results did not demonstrate any increase in brain activity for nominative and accusative case conditions compared with the genitive case condition. Of course, because this result is statistically negative, we cannot conclude that there is no brain response for the common processing of nominative and accusative cases as structural case processing. However, at least, we found no supportive evidence for such theoretical distinctions in the human brain.

In contrast, our results showed that genitive case processing requires greater brain activity than nominative and accusative case processing in the left triangular and orbital part of the inferior frontal gyrus, the posterior part of the middle temporal gyrus, and the supplementary motor area (Fig. 2 and Table 3). At this time, there are two possible interpretations for this finding. The first is that the theoretical distinction between structural (nominative and accusative) and inherent cases (genitive case), as described in the Introduction, may be supported by our results. However, while we found greater brain activity for inherent case processing than for structural case processing, activity was not greater for structural case processing than for inherent case processing. If a specific cognitive process for structural cases exists, a brain region more activated for these two cases than for the genitive case should exist as well. However, we’ve found no such region. Also, although brain activity was weaker than in the genitive case, the nominative and accusative cases activated all ROIs which showed different activity between the genitive and nominative/accusative cases (see Figure 3). Hence, the fMRI data we observed here might not be due to the theoretical distinction between inherent case processing and structural case processing.

The second possible interpretation involves different structural complexity in processing the genitive and the nominative/accusative cases. Since the genitive case is embedded in a complex NP ([_ ’s N]), it may require a greater processing load from some complex structure than the nominative and accusative cases. In previous neuroimaging studies, structural or syntactic complexity activated the left inferior frontal gyrus and the lateral posterior part of the temporal lobe [10], [17], [18], which is similar to our results. Hence, the genitive case should show greater brain activation for the processing load than for the nominative and accusative cases. Actually, this interpretation is also supported by behavioral data which revealed that reaction times in the genitive case condition were longer than those in the other two case conditions (see Results). However, across conditions, our analysis excluded any possible effect from a difference in reaction times which would be reflected in the processing effort or load. We dealt with reaction times as a confounding covariate in our statistical analysis of fMRI data. Also, we set the statistical threshold at 0.05, corrected for multiple comparisons (FWE), which is known to be a very strict threshold for the statistical analysis of fMRI data. Thus, the effect of a difference in reaction times should be excluded from our fMRI results, suggesting that the greater brain activation for genitive case processing than for nominative and accusative case processing is not caused by an effort to process genitive case. Of course, since it is still unclear whether the effect of processing load has been completely excluded from our main results, we believe that the latter interpretation is more plausible. But, since this second possible interpretation also has some limitations/problems, further study is necessary to clarify case processing.

From different point of view, there is another potential interpretation of our results. That is based on the notion of semantic/thematic role, rather than the notion of case. Any NP in a sentence has its own thematic role based on the verb (or the noun, if the sentence contains one) in the sentence. For example, in sentences such as *She saw him* (in English), the subject *she* has an agent role (in the sense that it functions as an agent of the action of seeing), and the object *him* has a thematic role (in the sense that it is the target/theme of seeing). Likewise, in examples such as *his car*, *his* has a possessor role. In most cases, the types of case and the thematic roles have a one-to-one relation. Therefore, in *She saw him* and *his car* (in English), *she* is the nominative case and has an agent role, *him* is the accusative case and has a thematic role, and *his* is the genitive case and has a possessor role. Hence, one may point out that our results can be interpreted not only by the notion of case but also by the notion of thematic roles. However, a thematic-role based analysis would not be able to correctly predict the results obtained in this study. This is because no clear distinction can be theoretically made between the NPs with the nominative and accusative cases and those with the genitive case, given the fact that in expressions such as *his lecture*, for instance, although *his* ought to have an agent role rather than a possessor role, it is a genitive case. Consequently, we can conclude that the observed fMRI results are caused by a difference in case processing, *per se*. From a structural perspecive, given that the brain regions identified in our study have been reported to be involved in grammatical/syntactic processing that includes case processing [10], [11], [12], [13], [15], [17], [19]–[24], the location of the observed brain activity is in line with these previous studies.

In the current study, to examine how case is processed in our brain, we used fMRI and scanned the brain activity of native English speakers during English case processing tasks. The results showed that, while processing of all cases activates the left triangular and orbital parts of the inferior frontal gyrus, the posterior part of the middle temporal gyrus, and the supplementary motor area, genitive case processing activates these two regions more than nominative and accusative case processing does. Since the effect of the difference in behavioral performance among these three cases is excluded from brain activation data, the observed difference in brain activations would be due to the different processing patterns among cases. The different brain activations between genitive case processing and nominative/accusative case processing may be due to the difference in structural complexity between them. However, there still remains a possibility that the case processing mechanisms in English observed in the current study cannot be generalized for all natural languages. One of the main reasons why generalization may be difficult is that the way case is indicated in a sentence differs among languages. For example, as described in Introduction, while some languages use case particles (e.g., Japanese, Korean), others use word order or morphological case markers that inflect pronouns (e.g., Chinese, English). Such a difference may cause different mental processes for case, and may show different brain activation patterns [25], [26]. Further study is necessary to examine whether a difference in case processing in the brain exists between the two types of languages.
